# Impact of the COVID-19 pandemic on the epidemiology of out-of-hospital cardiac arrest: a systematic review and meta-analysis

**DOI:** 10.1186/s13613-021-00957-8

**Published:** 2021-12-07

**Authors:** Seth En Teoh, Yoshio Masuda, Darren Jun Hao Tan, Nan Liu, Laurie J. Morrison, Marcus Eng Hock Ong, Audrey L. Blewer, Andrew Fu Wah Ho

**Affiliations:** 1 Yong Loo Lin School of Medicine, National University of Singapore, Singapore, Singapore; 2 Centre for Quantitative Medicine, Duke-NUS Medical School, Singapore, Singapore; 3 Division of Emergency Medicine, Department of Medicine, Li Ka Shing Knowledge Institute, St Michael’s Hospital, University of Toronto, Toronto, ON Canada; 4 Office C, Department of Emergency Medicine, Singapore General Hospital, 1 Outram Rd, Singapore, 169608 Singapore; 5 Health Services & Systems Research, Duke-NUS Medical School, Singapore, Singapore; 6 Department of Family Medicine and Community Health, and Department of Population Health Sciences, Duke University School of Medicine, Durham, NC USA; 7 Pre-Hospital and Emergency Research Centre, Duke-NUS Medical School, Singapore, Singapore

**Keywords:** Cardiac arrest, COVID-19, Pandemic, Coronavirus, Emergency medical services, Resuscitation, Ambulance, Sudden cardiac death, Out of hospital, OHCA, Epidemiology

## Abstract

**Background:**

The coronavirus disease 2019 (COVID-19) pandemic has significantly influenced epidemiology, yet its impact on out-of-hospital cardiac arrest (OHCA) remains unclear. We aimed to evaluate the impact of the pandemic on the incidence and case fatality rate (CFR) of OHCA. We also evaluated the impact on intermediate outcomes and clinical characteristics.

**Methods:**

PubMed, EMBASE, Web of Science, Scopus, and Cochrane Library databases were searched from inception to May 3, 2021. Studies were included if they compared OHCA processes and outcomes between the pandemic and historical control time periods. Meta-analyses were performed for primary outcomes [annual incidence, mortality, and case fatality rate (CFR)], secondary outcomes [field termination of resuscitation (TOR), return of spontaneous circulation (ROSC), survival to hospital admission, and survival to hospital discharge], and clinical characteristics (shockable rhythm and etiologies). This study was registered in the International Prospective Register of Systematic Reviews (PROSPERO) (CRD42021253879).

**Results:**

The COVID-19 pandemic was associated with a 39.5% increase in pooled annual OHCA incidence (*p* < 0.001). Pooled CFR was increased by 2.65% (*p* < 0.001), with a pooled odds ratio (OR) of 1.95 for mortality [95% confidence interval (95%CI) 1.51–2.51]. There was increased field TOR (OR = 2.46, 95%CI 1.62–3.74). There were decreased ROSC (OR = 0.65, 95%CI 0.55–0.77), survival to hospital admission (OR = 0.65, 95%CI 0.48–0.89), and survival to discharge (OR = 0.52, 95%CI 0.40–0.69). There was decreased shockable rhythm (OR = 0.73, 95%CI 0.60–0.88) and increased asphyxial etiology of OHCA (OR = 1.17, 95%CI 1.02–1.33).

**Conclusion:**

Compared to the pre-pandemic period, the COVID-19 pandemic period was significantly associated with increased OHCA incidence and worse outcomes.

**Supplementary Information:**

The online version contains supplementary material available at 10.1186/s13613-021-00957-8.

## Introduction

The coronavirus disease 2019 (COVID-19) pandemic, caused by the emergent novel severe acute respiratory syndrome coronavirus 2 (SARS-CoV-2), has had a far-reaching public health impact of global proportions [1, 2]. This has influenced the epidemiology of important diseases, not only through the direct impact of COVID-19 infections but also through indirect effects on health services delivery, access to care, and health-seeking behaviors [3].

In particular, the impact of the pandemic on out-of-hospital cardiac arrest (OHCA) epidemiology is of tremendous public health and scientific interest [4]. Not only does OHCA exert a large disease burden in many communities, its care processes also reflect the efficient integration of bystander care, ambulance care, and hospital care within health systems [5]. A growing body of anecdotal and scientific reports from some parts of the world have suggested that the COVID-19 pandemic was associated with changes in incidence, care processes, and clinical outcomes. For example, a population-based observational study in Paris, France, reported that the maximum weekly OHCA incidence had nearly doubled from 13.42 to 26.64 per million population during the pandemic period (March–April, 2020) compared to the pre-pandemic period (March–April, 2012–2019) [6]. However, reports from other parts of the world did not find a similar association [7, 8].

While some of the negative effects of the pandemic on incidence and outcomes were likely mediated through COVID-19 infections (especially OHCA of respiratory nature), it is likely that there are multifactorial drivers of these trends [9, 10]. These may include the interruption of primary care for chronic diseases, reduced willingness to seek early treatment for acute symptoms leading to increased risk of OHCA, as well as worsened prognosis due to less efficient bystander, ambulance, and hospital interventions for OHCA [11–15]. These treatment processes may have, in turn, been adversely affected by reduced bystander willingness to render life-saving interventions [16, 17], altered airway management protocols [18], or even overwhelmed healthcare facilities [19].

Given the rapidly evolving nature of this field, we aimed to perform an updated systematic review and meta-analysis on the impact of the COVID-19 pandemic on the incidence, characteristics, and clinical outcomes of OHCA. The increased geographical representation among our included studies lends generalizability to our findings. In addition, the larger overall sample size included in our study provides greater statistical power in support of the effects of COVID-19 on OHCA and related outcomes. Collectively, these add certainty to our conclusions. We also aimed to perform pooled estimates of OHCA incidence via a meta-analysis of proportions, which are important in contextualizing these data to the general population and ultimately useful in assessing the overall disease burden of OHCA during the pandemic period.

We primarily hypothesized that compared to the pre-pandemic period, the COVID-19 pandemic period was associated with increased incidence and case fatality rate (CFR) of OHCA. We further postulated that the rates of intermediate clinical outcomes (return of spontaneous circulation [ROSC], survival to hospital admission, and survival to hospital discharge) decreased during the pandemic. Finally, we postulated that there was a change in the etiologies of OHCA during the pandemic as well as a decline in the rate of shockable rhythm as the initial presenting rhythm. A consolidated understanding of the impact of the COVID-19 pandemic on OHCA epidemiology will inform future pandemic preparedness and response strategies.

## Methods

This systematic review and meta-analysis adhered to the Preferred Reporting Items for Systematic Reviews and Meta-Analyses (PRISMA) guidelines [20]. It was registered in the International Prospective Register of Systematic Reviews (PROSPERO) (CRD42021253879).

### Search strategy

The search strategy was developed in consultation with a medical information specialist. We utilized the MeSH term “heart arrest” and the non-MeSH terms “sudden cardiac arrest, sudden cardiac death, out of hospital cardiac arrest, out-of-hospital cardiac arrest, cardiac arrest, OHCA, OOHCA, covid-19, coronavirus, and SARS-CoV-2” (Additional file 1: Appendix I). We performed a literature search with these search terms in five bibliographic databases from inception to May 3, 2021: PubMed, EMBASE, Web of Science, Scopus, and The Cochrane Central Register of Controlled Trials (CENTRAL) in The Cochrane Library. References of relevant sources were hand searched to identify additional relevant studies.

### Selection criteria

All studies were filtered through the following inclusion criteria: (A) patients with OHCA during the COVID-19 pandemic and (B) articles that reported any of the following outcomes and clinical characteristics: incidence of OHCA, mortality, field TOR, ROSC, survival to hospital admission, survival to hospital discharge, shockable rhythm, and etiology of OHCA.

The exclusion criteria were as follows: (A) articles not written in the English language, (B) articles that did not utilize a historical control (comparing outcomes prior to and during the pandemic), (C) case reports, and (D) editorials.

We did not attempt to collect non-peer reviewed data for our study on the basis that unpublished studies may be of lower methodological quality than published ones (e.g., unpublished studies were less likely to conceal intervention allocation adequately and to blind outcome assessments) [21]. This problem is noted to be especially pertinent for COVID-19-related studies published in abstract-form and pre-print services [22].

The hierarchical selection of titles, abstracts, and full manuscripts was conducted by two independent reviewers, SET and YM. Subsequently, the web-based platform Rayyan QCRI was utilized to deconflict selected articles in a blinded manner [23]. Disagreements were resolved after consensus with the senior author, AFWH.

### Data abstraction and quality assessment

Three authors (SET, YM, and DJHT) independently abstracted data using a predefined spreadsheet (Microsoft Corp, New Mexico, United States). Conflicts with data collection were resolved after consensus with the senior author, AFWH. Study characteristics abstracted included the name of first author, year of publication, country and city of study population, study design, study population, date cut-offs for time periods of (i) pre-COVID-19 and (ii) COVID-19, and sample sizes for time periods of (i) pre-COVID-19 and (ii) COVID-19. Moreover, we abstracted patient characteristics, including age, percentage who were male, percentage with shockable rhythm, and percentage with different etiologies of OHCA, namely medical, traumatic, drowning, overdose, and asphyxial. Lastly, we abstracted outcomes data: incidence, mortality, field TOR, ROSC, survival to hospital admission, and survival to hospital discharge. If data were unclear, we contacted the corresponding author of the publication via email for further clarification. We manually ensured that the included studies did not utilize overlapping datasets and after thorough inspection, we did not discover any evidence of these.

The Newcastle–Ottawa Scale [24] was used by two independent reviewers (SET and YM) to evaluate the risk of bias within the included studies [6–8, 25–41]. The scale included eight items, and possible scores ranged from 0 to 9. Studies with a score of seven or more were considered high quality.

### Calculation of annual OHCA incidence

Incidence estimates were abstracted only from studies that used a population-based data source, allowing the inference of population parameters. Where studies did not directly report incidence, we manually computed incidence if the studies provided data on case counts and the corresponding population-at-risk (i.e., coverage of the data source). This assumed that population-at-risk remained constant over the study period, and that each unique person could contribute only one OHCA episode. We used the reference period of each respective study to standardize these to annual incidence per 100,000 population. Incidence estimates of individual communities were weighted and averaged according to the size of the population-at-risk. This approach was previously utilized by Berdowski et al. in a systematic review of OHCA incidence [42].

### Statistical analysis

Data analyses were performed using the *meta 4.18–0* and *metafor 2.4–0* packages in R 3.6.3 (R Foundation for Statistical Computing, Vienna, Austria). Meta-analyses of proportions were conducted for the outcomes of annual OHCA incidence and CFR. Meta-analyses were performed for the characteristics: shockable rhythm and etiologies of OHCA (medical, traumatic, and asphyxial) and outcomes: mortality, field TOR, ROSC, survival to hospital admission, and survival to hospital discharge.

In the meta-analyses of proportions, data for the outcomes of annual OHCA incidence and CFR were transformed using the Freeman-Tukey double arcsine method [43]. The transformed data were then pooled through an inverse variance methodology, before back-transformation into normalized proportions. The DerSimonian-Laird estimator was used as our between-study variance estimator, and a random-effects model was employed to estimate the pooled study estimates due to marked study heterogeneity. Results were then presented in forest plots, with relevant outcomes reported as proportions with 95% confidence intervals (95%CI). Comparisons between the outcomes of annual OHCA incidence and CFR for pre-COVID-19 and COVID-19 periods were performed with two-proportion z-tests. Box plots were used to represent these results.

For other outcomes, fixed- and random-effects models were used in conjunction with the Sidik-Jonkman estimator and Mantel–Haenszel method to estimate the pooled effects of COVID-19, depending on the presence of substantial between-study heterogeneity. Forest plots displayed pooled odds ratios (OR) and 95%CI for mortality, field TOR, ROSC, survival to hospital admission, survival to hospital discharge, shockable rhythm, and etiologies of OHCA. Two-tailed statistical significance was set at *p* value ≤ 0.05. The I^2^ statistic was used to quantify statistical heterogeneity [44]. Whenever there was substantial statistical heterogeneity (*I*^2^ > 50%), we evaluated for outliers by performing a set of case deletion diagnostics to identify influential studies and subsequent leave-one-out sensitivity analyses. Publication bias was evaluated via visual evaluation of funnel plots and Egger’s regression.

## Results

### Literature retrieval

**Fig. 1:**
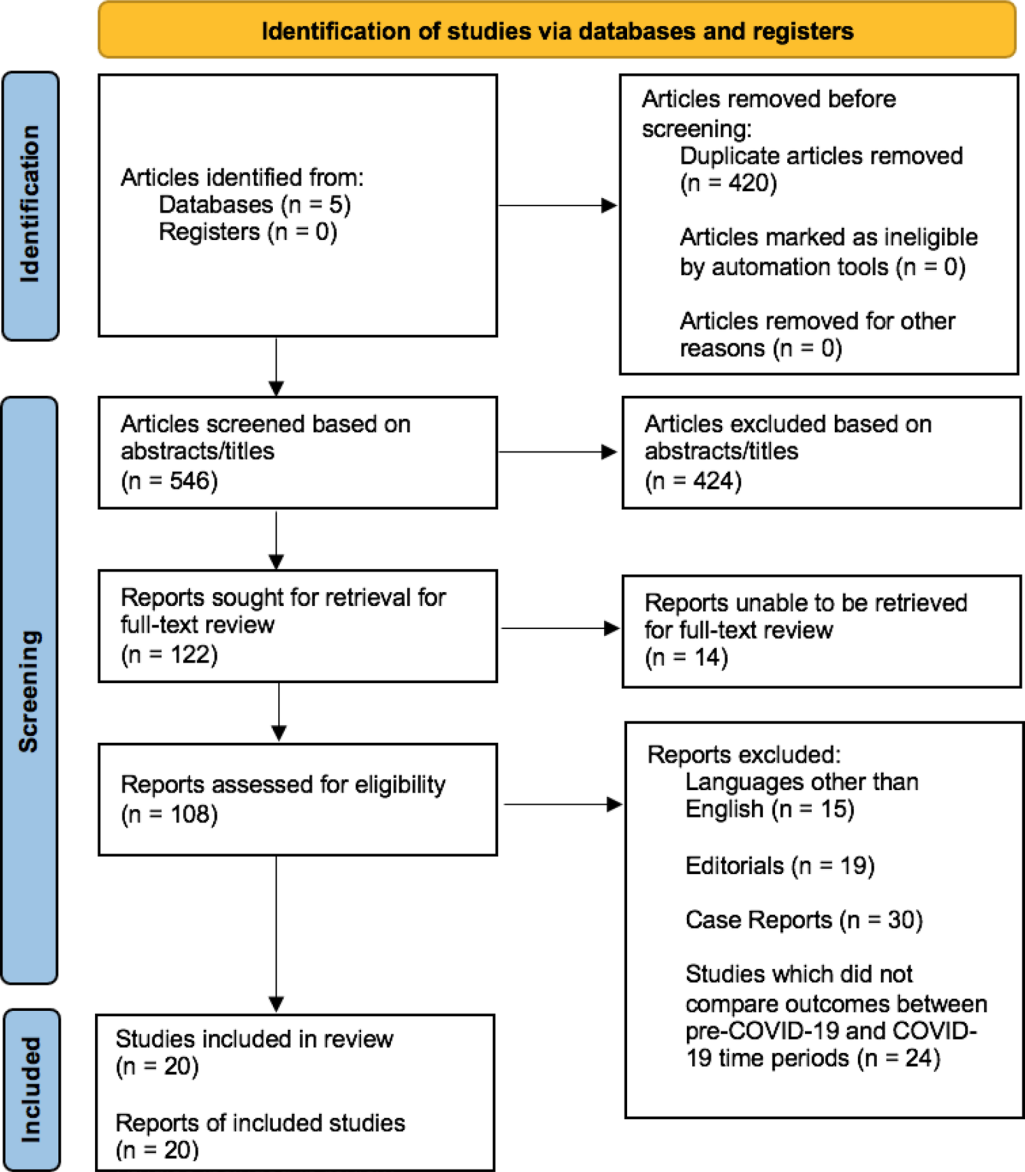
PRISMA-P 2020 flow diagram for study selection

The database search yielded 966 articles. After removal of duplicates, 546 articles were screened on the basis of their abstracts. After screening, 122 papers were sought for retrieval, of which 14 articles could not be retrieved. The resultant 108 full texts were reviewed, and 20 identified as meeting the selection criteria. The study selection process and reasons for excluding the 88 excluded studies were illustrated in the PRISMA-P 2020 Flow Diagram (Fig. 1).

### Characteristics of studies and risk of bias

The included studies originated from 10 countries (Australia, France, Italy, Korea, Singapore, Spain, Sweden, The Netherlands, United Kingdom, and United States of America). All studies were retrospective in study design.

There were a total of 67,815 patients included in the 20 studies, comprising 38,855 patients in the pre-COVID-19 period and 28,960 patients in the COVID-19 period. Study sample sizes ranged from 101 to 19,303 patients. Study characteristics and the summary of overall findings were summarized in Tables 1 and 2, respectively.

**Table 1:** Characteristics of Included Studies

Study	Location	Study Design^a^	Study Population	Time Period (i) Pre-COVID-19 (ii) COVID-19	Sample Size (i) Pre-COVID-19 (ii) COVID-19
Baert et al. 2020 [25]	France	Registry-based study	Adult and pediatric cases of presumed medical etiology (EMS-treated NR; Received resuscitation NR)	(i) March 1–April 31, 2019 (ii) March 1–April 31, 2020	(i) 1620 (ii) 1005
Baldi et al. 2020 [26]	Lombardy, Italy	Registry-based study	Adult and pediatric cases regardless of etiology (EMS-treated NR; Received resuscitation NR)	(i) February 21–April 20, 2019 (ii) February 21–April 20, 2020	(i) 321 (ii) 490
Ball et al. 2020 [7]	Victoria, Australia	Registry-based study	Adult cases regardless of etiology; EMS-treated and received resuscitation	(i) March 16–May 12, 2017–2019 (ii) March 16–May 12, 2020	(i) 1218 (ii) 380
Cho et al. 2020 [27]	Daegu, South Korea	Registry-based study	Adult cases of presumed medical etiology; EMS-treated and received resuscitation	(i) February 17–March 31, 2018 (ii) February 17–March 31, 2020	(i) 158 (ii) 171
Elmer et al. 2020 [28]	Pennsylvania, USA	Registry-based study	Adult cases regardless of etiology; EMS-treated (Received resuscitation NR^§^)	(i) January–February 2016–2020 (ii) March 1–May 25, 2020	(i) 12,252 (ii) 683
Lai et al. 2020 [29]	New York City, USA	Non-registry-based study	Adult cases regardless of etiology; EMS-treated and received resuscitation	(i) March 1–April 25, 2019 (ii) March 1–April 25, 2020	(i) 1336 (ii) 3989
Marijon et al. 2020 [6]	Paris, France	Registry-based study	Adult cases of non-traumatic etiology; EMS-treated (Received resuscitation NR)	(i) Weeks 12–17, 2012–2019 (ii) March 16–April 26, 2020	(i) 3052 (ii) 521
Ortiz et al. 2020 [30]	Spain	Registry-based study	Adult and pediatric cases regardless of etiology; EMS-treated (Received resuscitation NR)	(i) April 1–30, 2017 and February 1–March 31, 2018 (ii) February 1–April 30, 2020	(i) 1723 (ii) 1446
Paoli et al. 2020 [31]	Province of Padua, Italy	Non-registry-based study	Adult and pediatric cases regardless of etiology; EMS-treated (Received resuscitation NR^§^)	(i) March 1–April 30, 2019 (ii) March 1–April 30, 2020	(i) 206 (ii) 200
Sayre et al. 2020 [32]	Seattle and King County, USA	Registry-based study	Adult and pediatric cases regardless of etiology; EMS-treated (Received resuscitation NR^§^)	(i) January 1–February 25, 2019 (ii) February 26–April 15, 2020	(i) 530 (ii) 537
Semeraro et al. 2020 [33]	Bologna, Italy	Registry-based study	Adult cases regardless of etiology; EMS-treated and received resuscitation	(i) January 1–June 30, 2019 (ii) January 1–June 30, 2020	(i) 563 (ii) 624
Chan et al. 2021 [34]	27 States and multiple Counties, USA	Registry-based study	Adult cases of non-traumatic etiology; EMS-treated (Received resuscitation NR)	(i) March 16–April 30, 2019 (ii) March 16–April 30, 2020	(i) 9440 (ii) 9863
de Koning et al. 2021 [35]	Hollands-Midden, The Netherlands	Registry-based study	Adult cases regardless of etiology; EMS-treated (Received resuscitation NR)	(i) March 16–April 27, 2019 (ii) March 16–April 27, 2020	(i) 45 (ii) 56
Fothergill et al. 2021 [8]	London, UK	Registry-based study	Adult and pediatric cases regardless of etiology; EMS^*^-treated (Received resuscitation NR^§^)	(i) March 1–April 30, 2019 (ii) March 1–April 30, 2020	(i) 1724 (ii) 3122
Glober et al. 2021 [36]	Indiana (Marion County), USA	Registry-based study	Adult cases of non-traumatic etiology; EMS-treated (Received resuscitation NR)	(i) January 1–June 30, 2019 (ii) January 1–June 30, 2020	(i) 884 (ii) 1034
Lim et al. 2021 [37]	Singapore	Registry-based study	Adult cases regardless of etiology; EMS-treated (Received resuscitation NR)	(i) January 1–May 31, 2018–2019 (ii) January 1–May 31, 2020	(i) 1280 (ii) 1400
Mathew et al. 2021 [38]	Detroit, USA	Registry-based study	Adult cases of non-traumatic etiology; EMS-treated and received resuscitation	(i) March 10–April 30, 2019 (ii) March 10–April 30, 2020	(i) 180 (ii) 291
Nickles et al. 2021 [39]	Detroit (Macomb, Oakland, and Wayne Counties), USA	Registry-based study	Adult and pediatric cases of non-traumatic etiology; EMS-treated (Received resuscitation NR)	(i) January 1–May 31, 2019 (ii) January 1–May 31, 2020	(i) 1162 (ii) 1854
Sultanian et al. 2021 [40]	Sweden	Registry-based study	Adult and pediatric cases regardless of etiology; EMS-treated and received resuscitation	(i) January 1–March 16, 2020 (ii) March 16–July 20, 2020	(i) 930 (ii) 1016
Uy-Evanado et al. 2021 [41]	Oregon (Multnomah County) and California (Ventura County), USA	Registry-based study	Adult and pediatric cases regardless of etiology; EMS-treated and received resuscitation	(i) March 1–May 31, 2019 (ii) March 1–May 31, 2020	(i) 231 (ii) 278

*EMS* Emergency Medical Services, *UK *United Kingdom, *USA* United States of America, *NR* Not Reported, *COVID-19* coronavirus disease 2019

^a^Study designs for all included studies were multicentered and retrospective in nature

**Table 2:** Summary of Overall Findings

OHCA outcomes and characteristics	Parameters		Number of studies	Pooled OR (95% CI)	*P* value	*I*^2^ statistic
Primary outcomes	Annual Incidence^#^		10	N/A	< 0.001	N/A
	Case Fatality Rate^#^		11	N/A	< 0.001	N/A
	Mortality		11	1.95 (1.51–2.51)	0.0002	67%
Secondary outcomes	Termination of Resuscitation		5	2.46 (1.62–3.74)	0.0040	93%
	ROSC		15	0.65 (0.55–0.77)	< 0.0001	85%
	Survival to Hospital Admission		10	0.65 (0.48–0.89)	0.0122	87%
	Survival to Hospital Discharge		11	0.52 (0.40–0.69)	0.0004	67%
Characteristics	Shockable Rhythm		15	0.73 (0.60–0.88)	0.0024	70%
	Etiology	Medical	9	0.91 (0.60–1.37)	0.5922	93%
		Traumatic	7	0.68 (0.41–1.13)	0.1108	70%
		Asphyxial	5	1.17 (1.02–1.33)	0.0317	0%

*ROSC* Return of Spontaneous circulation, *CI* Confidence Interval, *OR* Odds Ratio, *N/A* not applicable, *OHCA* out-of-hospital Cardiac arrest

^#^*P* values were obtained from two-proportion z-tests comparing Pre-COVID-19 and COVID-19 pooled values

All studies achieved a score ranging from 7 to 9 on the Newcastle–Ottawa Scale, signifying high quality and low risk of bias for selection (Additional file 1: Table S1).

#### Primary outcomes: annual ohca incidence

Ten studies reported or provided sufficient data to calculate the annual OHCA incidence (per 100,000 population) (Additional file 1: Table S2) [6–8, 26, 31, 32, 35–37, 39]. Among them, de Koning et al. [35] reported the lowest annual OHCA incidence of 51 and 63 cases per 100,000 population in the pre-COVID-19 and COVID-19 time periods, respectively. Meanwhile, Glober et al. [36] reported the highest annual OHCA incidence of 183 and 214 cases per 100,000 population in the pre-COVID-19 and COVID-19 time periods, respectively. With the exception of Paoli et al. [31], all studies reported a trend of higher annual OHCA incidence during the COVID-19 period compared to the pre-pandemic period.

**Fig. 2:**
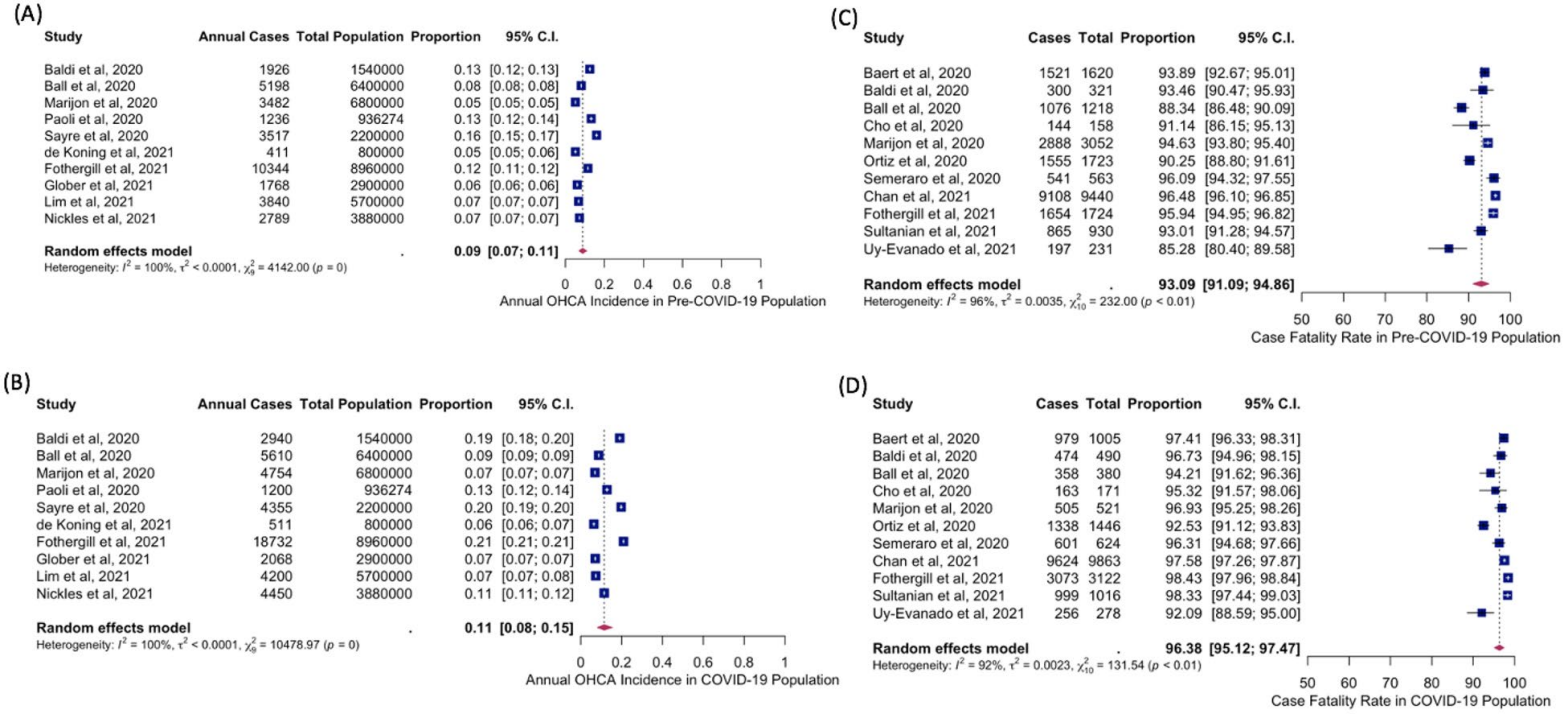
Forest plots of estimates from meta-analysis of proportions—**A** Annual OHCA incidence in Pre-COVID-19 time period. **B** Annual OHCA incidence in COVID-19 time period. **C** Case fatality rate in Pre-COVID-19 time period. **D** Case fatality rate in COVID-19 time period *COVID-19,* coronavirus disease 2019; *OHCA*, out-of-hospital cardiac arrest

The meta-analysis of proportions showed an annual OHCA incidence of 0.0860% or 86.0 cases per 100,000 population (34,511 cases out of 40,116,274 population) in the pre-COVID-19 period (95%CI 0.07–0.11%) with a heterogeneity of *I*^2^ = 100%, *p* < 0.01. In contrast, the annual OHCA incidence was 0.12% or 121.7 cases per 100,000 population (48,820 cases per 40,116,274 population) in the COVID-19 period (95%CI 0.08–0.15%) with a heterogeneity of *I*^2^ = 100%, p < 0.01 (Fig. 2A, B).

#### Primary outcomes: mortality and case fatality rate

Eleven studies provided sufficient data on mortality [6–8, 25–27, 30, 33, 34, 40, 41]. Mortality ranged from 85.3 to 98.4% across both pre-COVID-19 and COVID-19 time periods. During the pre-COVID-19 period, Uy-Evanado et al. reported the lowest mortality of 85.3%, while Chan et al. reported the highest mortality of 96.5% [34, 41]. In contrast, Uy-Evanado et al. reported the lowest mortality of 92.1%, while Fothergill et al. recorded the highest mortality of 98.4%, during the COVID-19 period [8, 41]. Overall, all studies reported higher mortality in the COVID-19 period compared to the pre-pandemic period.

**Fig. 3:**
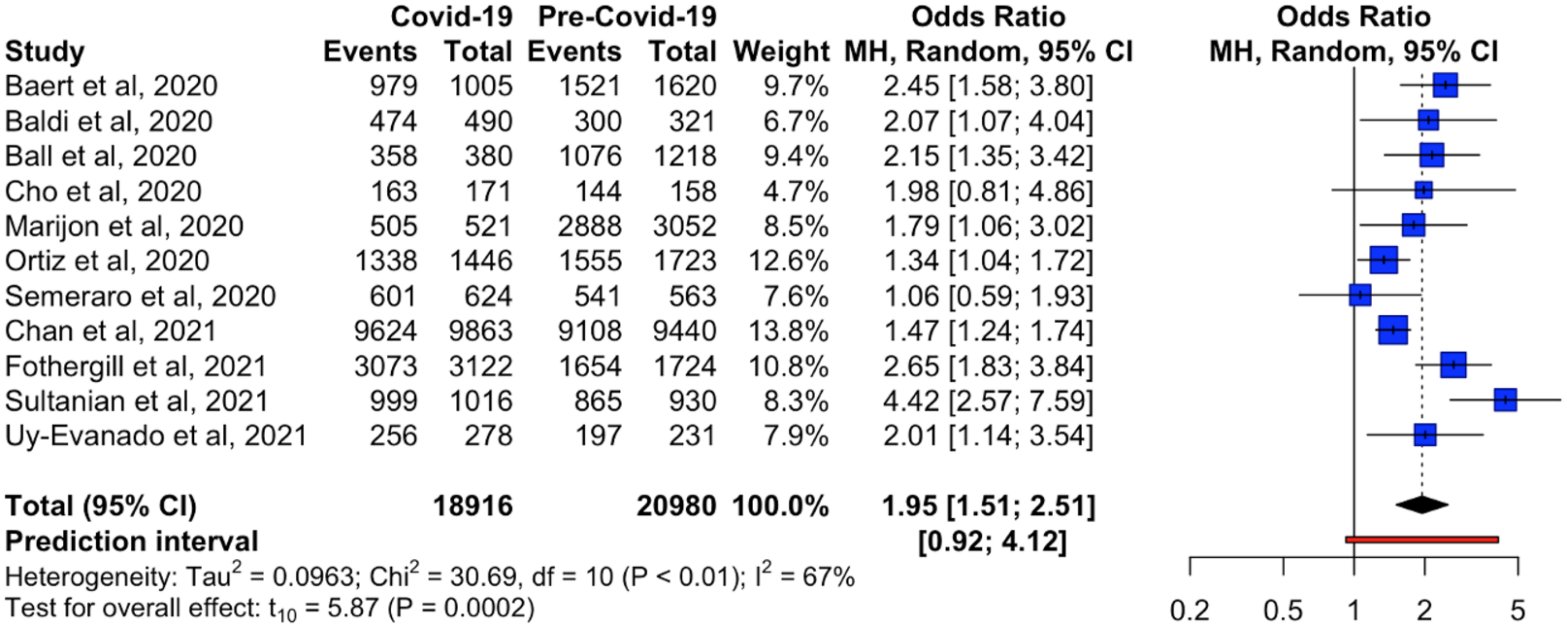
Forest plot of reported estimates for the primary outcome of mortality among patients with out-of-hospital cardiac arrest

The odds of mortality were significantly higher during the COVID-19 period as compared to the pre-COVID-19 period (OR = 1.95, 95%CI 1.51–2.51, *p* = 0.0002, *I*^2^ = 67%) (Fig. 3).

A separate meta-analysis of proportions for the outcome of CFR revealed a pooled rate of 93.09% in the pre-COVID-19 time period (95%CI 91.09–94.86%). Between-study heterogeneity was observed at *I*^2^ = 96%, *p* < 0.01. In the COVID-19 period, CFR was 96.38% (95%CI 95.12–97.47%) with a heterogeneity of *I*^2^ = 92%, *p* =  < 0.01 (Fig. 2C, D).

Difference in Pooled Estimates of COVID-19 and Pre-COVID-19 Time Periods for Annual OHCA Incidence and Case Fatality Rate.

**Fig. 4:**
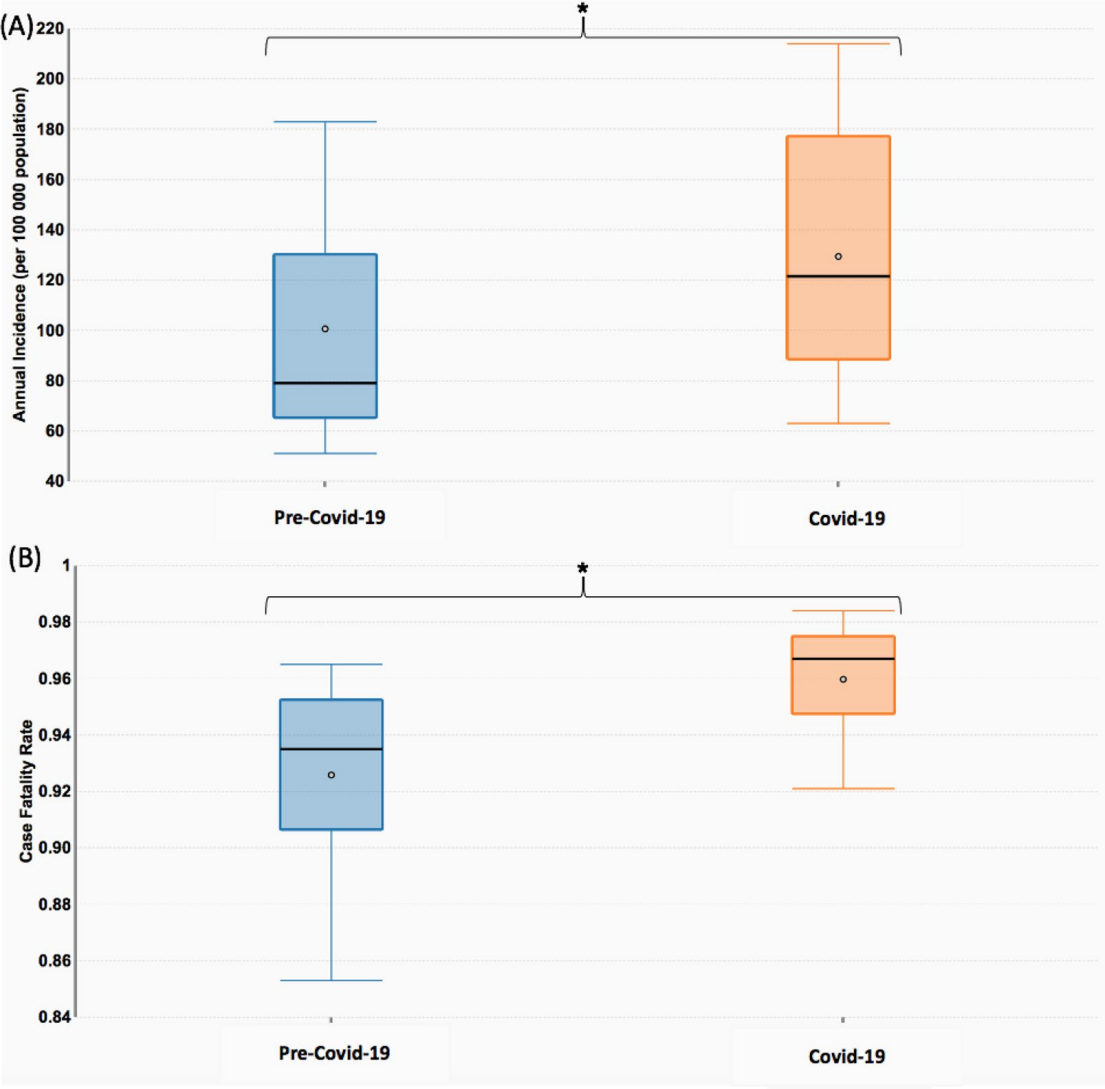
Box plots stratifying estimates for Pre-Covid-19 and COVID-19 time periods for **A** Annual OHCA Incidence. **B** Case Fatality Rate. Two-proportion z-tests were statistically significant (*p* < 0.001) for both outcomes, as represented by asterisks (*). *COVID-19* coronavirus disease 2019, *OHCA* out-of-hospital cardiac arrest

Comparing the pooled estimates for annual OHCA incidence and CFR for pre-COVID-19 and COVID-19 time periods, two-proportion z-tests revealed significant differences between pre-COVID-19 and COVID-19 periods for the outcomes of annual OHCA incidence (39.5% increase, p < 0.001) and CFR (2.65% increase, p < 0.001), as illustrated in Fig. 4.

#### Secondary outcomes

##### Termination of resuscitation in the field

Five studies reported the outcome of field TOR [8, 26, 29, 34, 38]. The percentage of population experiencing field TOR ranged from 35.6 to 89.5% across intervals of the pre-COVID-19 and COVID-19 time periods. All studies reported a trend of higher percentage of patients with field TOR during the COVID-19 period as compared to the pre-pandemic period.

**Fig. 5:**
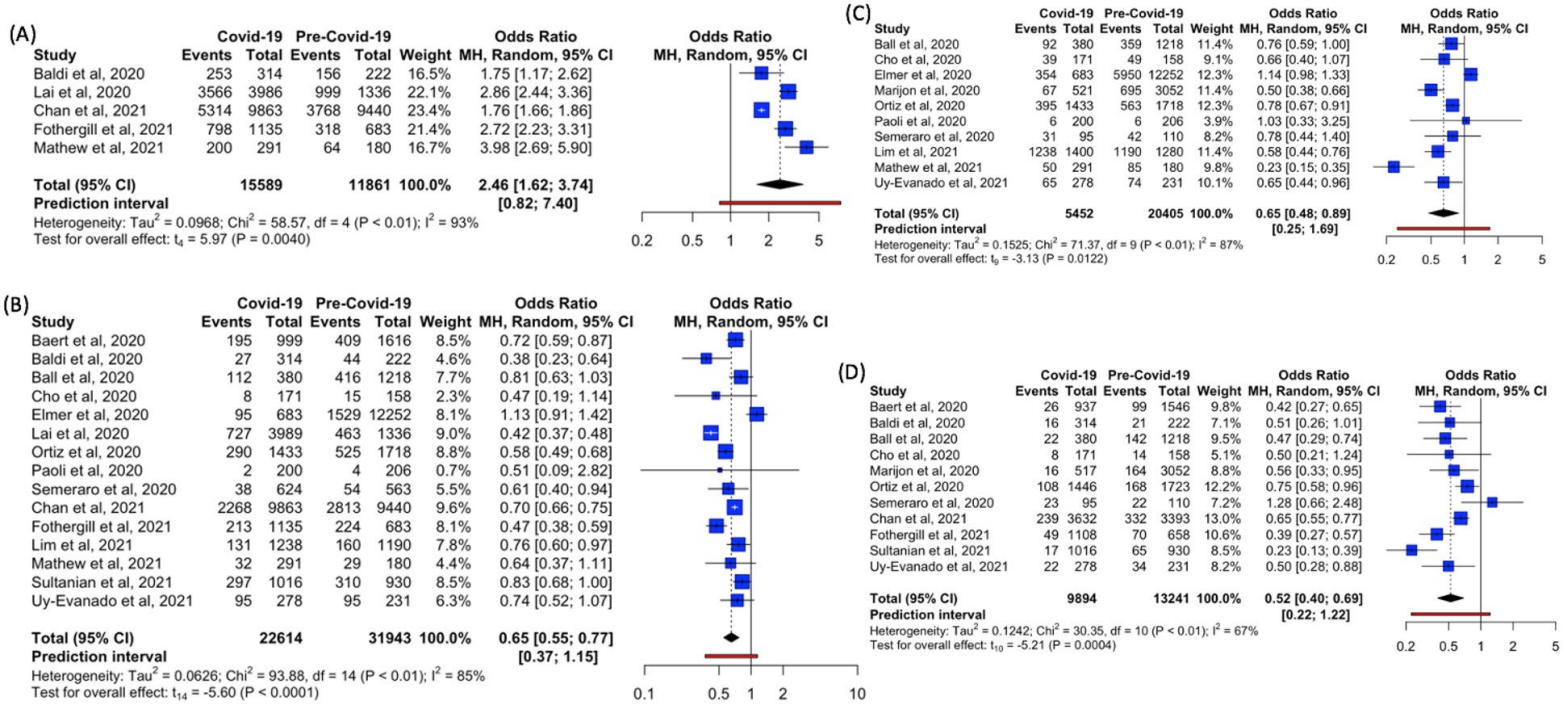
Forest plots of reported estimates for the secondary outcomes—**a** termination of resuscitation in the field. **B** Return of spontaneous circulation. **C** Survival to hospital admission. **D** Survival to Hospital discharge

Meta-analysis showed significantly higher odds of field TOR during the COVID-19 period as compared to the pre-COVID-19 period (OR = 2.46, 95%CI 1.62–3.74, p = 0.0040, I^2^ = 93%) (Fig. 5A).

##### Return of spontaneous circulation

Fifteen studies reported the outcome of ROSC [7, 8, 25–31, 33, 34, 37, 38, 40, 41]. The percentage of population experiencing ROSC ranged from 1.0 to 41.1% across intervals of the pre-COVID-19 and COVID-19 time periods. Almost all studies (except Elmer et al. [28]) reported a lower percentage of population with ROSC during the pandemic as compared to before the pandemic.

Meta-analysis showed significantly lower odds of ROSC during the COVID-19 time period as compared to the pre-COVID-19 period (OR = 0.65, 95%CI 0.55–0.77, p < 0.0001, I^2^ = 85%) (Fig. 5B).

##### Survival to hospital admission

Ten studies reported the outcome of survival to hospital admission [6, 7, 27, 28, 30, 31, 33, 37, 38, 41]. With the exception of Elmer et al. [28] and Paoli et al. [31], all studies reported a lower percentage of survival to hospital admission in the COVID-19 period relative to during the pre-COVID-19 period.

Meta-analysis showed significantly lower odds of survival to hospital admission in the COVID-19 period as compared to the pre-COVID-19 period (OR = 0.65, 95%CI 0.48–0.89, *p* = 0.0122, *I*^2^ = 87%) (Fig. 5C).

##### Survival to hospital discharge

Eleven studies reported the outcome of survival to hospital discharge [6–8, 25–27, 30, 33, 34, 40, 41]. Almost all studies (except Semeraro et al. [33]) reported a lower percentage of survival to hospital discharge in the COVID-19 period relative to during the pre-COVID-19 period.

Meta-analysis showed significantly lower odds of survival to hospital discharge during the COVID-19 period as compared to the pre-COVID-19 period (OR = 0.52, 95%CI 0.40–0.69, *p* = 0.0004, *I*^2^ = 67%) (Fig. 5D).

#### Clinical characteristics

##### Shockable rhythm

Fifteen studies provided data for the characteristic of shockable rhythm [6–8, 25–27, 29, 30, 33–35, 37, 38, 40, 41]. The percentage who had shockable rhythm ranged from 3.6 to 40% across intervals of the pre-COVID-19 and COVID-19 time periods. Apart from Semeraro et al. [33], all studies reported a trend of lower percentage with shockable rhythm in the COVID-19 period as compared to the pre-COVID-19 period.

**Fig. 6:**
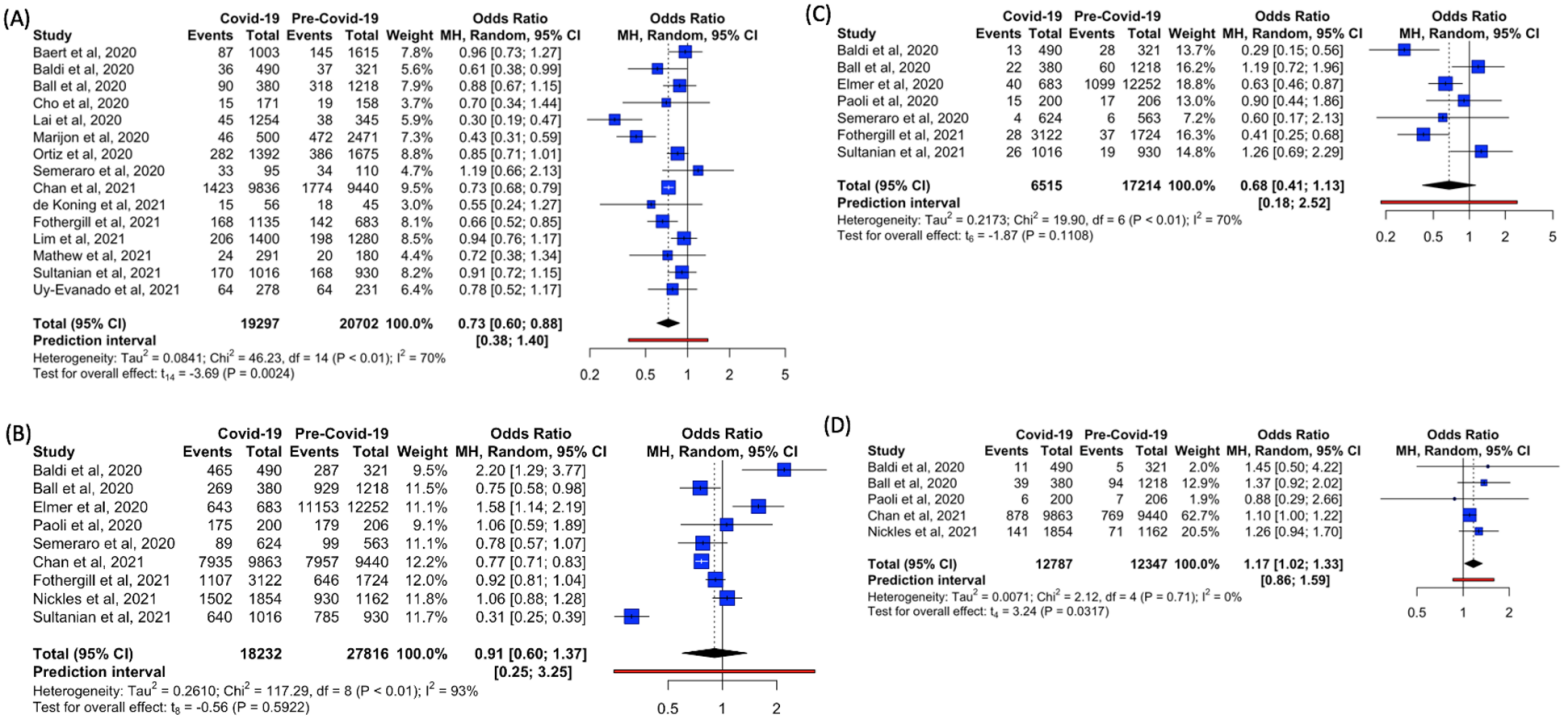
Forest plots of reported estimates for clinical characteristics of patients with out-of-hospital cardiac arrest—**A** Shockable rhythm. **B** Medical etiology. **C** Traumatic etiology. **D** Asphyxial etiology

Meta-analysis showed significantly lower odds of shockable rhythm in the COVID-19 period as compared to the pre-COVID-19 period (OR = 0.73, 95% CI 0.60–0.88, *p* = 0.0024, *I*^2^ = 70% (Fig. 6A).

##### Etiology

Nine [7, 8, 26, 28, 31, 33, 34, 39, 40], seven [7, 8, 26, 28, 31, 33, 40], and five [7, 26, 31, 34, 39] studies reported data for medical, traumatic, and asphyxial etiologies of OHCA, respectively (Additional file 1: Table S3). The majority of patients experienced OHCA from medical etiology.

Meta-analysis showed no difference in medical (OR = 0.91, 95%CI 0.60–1.37, *p* = 0.592, *I*^2^ = 93%) and traumatic etiologies (OR = 0.68, 95%CI 0.41–1.13, *p* = 0.1108, *I*^2^ = 70%) in both COVID-19 and pre-COVID-19 periods. There were significantly higher odds of asphyxial etiology (OR = 1.17, 95%CI 1.02–1.33, *p* = 0.0317, *I*^2^ = 0%) for OHCA in the COVID-19 period as compared to the pre-COVID-19 period (Figs. 6B–D). We did not analyze drowning and overdose etiologies of OHCA due to paucity of data.

### Sensitivity analyses

Sensitivity analyses on the influence of outliers were performed as there was substantial statistical heterogeneity observed in almost all outcomes and clinical characteristics (except asphyxial etiology). Potential outliers were first screened on visual inspection of their confidence intervals, followed by influential analyses using influential diagnostic plots and Baujat plots (Additional file 1: Figures S2–S25). Applying this approach on each clinical characteristic as well as the primary and secondary outcomes, none of the estimates were substantially changed in direction or magnitude, with the exception of survival to hospital admission where the direction was preserved, but the magnitude of effect was increased by 12%. The revised estimates on sensitivity analyses were as follows: mortality (OR = 1.79 [95%CI 1.46–2.20, *p* = 0.0001, *I*^2^ = 51%] after excluding Sultanian et al. [40]), field TOR (OR = 2.73 [95%CI 1.67–4.47, *p* = 0.00074, *I*^2^ = 65%] after excluding Chan et al. [34]), ROSC (OR = 0.68 [95%CI 0.59–0.80, *p *= 0.0001, *I*^2^ = 72%] after excluding Lai et al. [29]), survival to hospital admission (OR = 0.73 [95% CI 0.59–0.90, *p* = 0.0085, *I*^2^ = 80%] after excluding Mathew et al. [38]), survival to hospital discharge (OR = 0.57 [95%CI 0.45–0.71, *p* = 0.0003, *I*^2^ = 51%] after excluding Sultanian et al. [40]), shockable rhythm (OR = 0.77 [95% CI 0.67–0.89, *p* = 0.0017, *I*^2^ = 57%] after excluding Lai et al. [29]), medical etiology (OR = 1.02 [95%CI 0.76–1.38, *p* = 0.8768, *I*^2^ = 83%] after excluding Sultanian et al. [40]), and traumatic etiology (OR = 0.77 [95%CI 0.48–1.24, *p* = 0.2172, *I*^2^ = 62%] after excluding Baldi et al. [26]).

### Publication bias

The funnel plots were based on chosen primary and secondary outcomes with the highest number of studies (mortality and ROSC respectively). These plots revealed no visual asymmetry, hence suggesting the absence of publication bias (Additional file 1: Figure S1). This was supported by non-significant Egger’s regression tests (*p* = 0.09128 and *p* = 0.750 respectively).

## Discussion

In this systematic review and meta-analysis, the COVID-19 pandemic was likely associated with a significant 39.5% increase in pooled incidence and higher CFR with nearly doubled odds of mortality among cases. There was a decreased rate of shockable rhythm as the initial presenting rhythm, a decrease in rates of ROSC, survival to hospital admission, and survival to hospital discharge, and an increase in field TOR rate.

The increased incidence during the pandemic was consistently reported in all studies, except in Paoli et al.’s [31], which was inconclusive for change in incidence. Paoli et al.’s research letter was limited by a short follow-up period and resultant small sample size accumulated, which could have resulted in a false negative effect on incidence [31]. The increase in pooled incidence was sizeable at 39.5% which implied a substantially increased disease burden. The reasons for this increase were not explicitly investigated in any of these studies. It was likely that the increased population risk was multifactorial and mediated through both direct and indirect pathways. The direct impacts of the coronavirus included that of severe respiratory failure leading to hypoxic causes of arrest, venous thromboembolism, and also through cardiac involvement manifesting as myocarditis, acute coronary thrombosis, and arrhythmias [45, 46].

The same increase in incidence was observed in countries that had successfully limited the community spread of the COVID-19 virus during the study period, such as Singapore and Victoria, Australia [7, 37]. One of these indirect effects may be attributed to changes in health-seeking behavior, such as patients being reluctant to seek help for acute symptoms until the untreated illness progresses to a severe stage and manifests as OHCA [12, 47, 48]. There were reports of delayed presentations for acute myocardial infarction and heart failure during the pandemic reported around the world [49, 50]. In other cases, the disruption (or deprioritization) of “non-essential” health services provision during the pandemic may have adversely affected the ongoing management of chronic disease, leading to increased individual-level risk [6, 51]. In addition, independent of the reluctance to seek help, patients with severe acute illness may have deteriorated due to reduced access to emergency health services, such as emergency department overcrowding and delayed ambulance response time [17, 19, 52]. The relative importance of each causal pathway is likely to differ between cities, as our study found that OHCA of asphyxial etiology was increased in some, but not all studies.

The second significant finding was that of worsened clinical outcomes for OHCA during the pandemic. Of note, the pooled odds of mortality were double those of historical controls. We postulate that this was attributed to pandemic-related changes affecting each link in the chain of survival [53, 54]. Social distancing measures across the world may have inadvertently reduced the probability that an arrest occurred in a public setting, in the proximity of a bystander, to deliver bystander cardiopulmonary resuscitation, early defibrillation, early recognition, and early activation of emergency medical services (EMS) [55]. Concerns of contracting a communicable virus also adversely affected the willingness-to-help [56]. Finally, the ability of emergency health services (both pre-hospital and hospital) to deliver high quality care may have been compromised due to immensely stretched resources at a time when extensive resources were diverted to care for COVID-19 patients [19]. Of interest was the finding that there was substantial heterogeneity reflecting that the effects of the pandemic on OHCA outcomes differed greatly between studies. Some cities, such as London, UK, experienced little increase in mortality despite a large increase in incidence [8]. This was possibly a reflection of differences in emergency preparedness and surge capacity in emergency health services and culture.

The reason for increased field TOR found in our study was most likely directly related to the decreased rates of ROSC and shockable rhythm as the initial presenting rhythm, which are two of three criteria of the Basic Life Support TOR rule [57]. The third criteria is EMS witnessed arrest, and although this event characteristic was not reported in the studies, it is likely that the rates of these arrests decreased, due to overwhelmed ambulance resources as well as the pandemic requirement for revised ambulance protocols. Another reason could be the delay of patient interventions as medical professionals are required to don personal protective equipment (PPE) at the scene prior to resuscitation [18].

We reported a decreased proportion of shockable rhythm during the pandemic. As cardiac etiology OHCAs are more likely to present with shockable rhythm, this finding may imply a shift in underlying etiology to non-cardiac etiologies. Our meta-analysis suggests that there was a significant shift to an asphyxial etiology of cardiac arrest during the pandemic. However, this finding should be reviewed with the caution that there are substantial variations in classifying etiologies used by each study.

There are several implications of our findings. The large impact of indirect effects of a viral pandemic on the epidemiology of other diseases has tremendous public health significance. The need to sensibly maintain a functional healthcare system to serve non-COVID health needs cannot be understated. While immense public and political attention are given to performance indicators for COVID-19 cases, health systems need to prevent deterioration in the care of other diseases. When pandemics become protracted, this impact may progressively worsen and become multifactorial. This necessitates that health systems operate with some excess capacity during non-pandemic times, through the pursuit of technologies and policies that increase efficiency. In the case of OHCA, which is resource intensive to care for (in terms of requirements for rapid response, intensive care unit beds, and ventilators), these patients compete directly with COVID-19 patients for many of the same resources. The need for pre-emptively planned and rehearsed operating protocols for emergency care systems (both pre-hospital and hospital) in a pandemic is paramount for high performance during such times. These include stockpiling of PPE, procedural confidence when encumbered by PPE, and clear policies on field TOR. There should also be clearly defined triggers for a health system to enter disaster mode, when the focus switches from “saving everyone” to “doing the best for the most.” The crucial need for pertinent public health messaging to appropriately utilize emergency care services is markedly exemplified in such pandemics.

Several limitations of our study need acknowledgement. Firstly, all studies utilized a before-after comparison design. This design is inherently susceptible to secular trends leading to biased estimates. For example, the increase in incidence found could simply be a reflection of an underlying increasing trend (such as an aging population) which would have been observed even without a pandemic. This means that the effect sizes could be over-estimated. Each study defined the COVID-19 and pre-COVID-19 time periods differently. The pandemic is likely to exert varying epidemiological pressures over time, and the effect sizes estimated are dependent on the cut-offs chosen. Heterogeneity in cut-off definition causes contamination of exposure, which often has the effect of under-estimating effect sizes. The lockdown periods enforced in the various countries could potentially have effects on OHCA and related outcomes. However, we were unable to abstract data solely from the lockdown periods, due to the lack of available data. The included studies originated from regions with existing OHCA registries, which meant that lower-income countries without established OHCA surveillance mechanisms were under-represented. Our study conclusions are hence not generalizable to lower-income countries, which may be disproportionately affected by the pandemic. We were unable to ascertain the quality of data collected during the pandemic period. The pandemic period may have affected surveillance and data quality processes [58]. Under-capture of cases during the pandemic period would lead to consequent under-estimation of incidence. There was moderate-to-high statistical heterogeneity in most random-effects models constructed, which was likely to be multifactorial, with both real and artifactual components. This was possibly attributed to the intrinsic differences in population demographics among included studies. Among our included studies, apart from the substantial variations in defining OHCA patients in the different registries, large differences in the organization of health systems across communities, including differences in policies of field TOR applied in each country during the pandemic period, could have also contributed to the wide variations in outcomes. We were unable to abstract these data from our included studies, owing to their paucity. Finally, one cannot discount the possibility that the observations were partly artifactual, specifically from publication bias which favored the publication of positive findings. This is a concern as there has been a proliferation of studies with high risk of bias owing to the exuberance of researchers and hastened editorial process [59]. However, our formal assessment for publication bias revealed no evidence of this among the included studies.

## Conclusion

The COVID-19 pandemic was likely associated with significant changes in OHCA epidemiology. Compared to the pre-pandemic period, the pandemic period seemed to be associated with increased incidence and CFR of OHCA. There were corresponding changes in intermediate outcomes and clinical characteristics.

## Supplementary Information

**Additional file 1: Appendix I**. Expanded Methods. **Table S1**. Newcastle-Ottawa Scale (NOS) for Risk of Bias Assessment of Studies Included in the Meta-Analysis. **Table S2**. Summary of Primary and Secondary Outcomes. **Table S3**. Characteristics of Patients with Out-of-Hospital Cardiac Arrest in the Pre-COVID-19 and COVID-19 Time Periods in the Included Studies. **Figure S1**. Funnel Plots based on (A) Mortality and (B) Return of Spontaneous Circulation. **Figure S2**. Influential Diagnostic Plot for Mortality. **Figure S3**. Baujat Plot for Mortality. **Figure S4**. Leave-One-Out Analysis For Mortality. **Figure S5**. Influential Diagnostic Plot for Termination of Resuscitation in the Field. **Figure S6**. Baujat Plot for Termination of Resuscitation in the Field. **Figure S7**. Leave-One-Out Analysis for Termination of Resuscitation in the Field. **Figure S8**. Influential Diagnostic Plot for Return of Spontaneous Circulation. **Figure S9**. Baujat Plot for Return of Spontaneous Circulation. **Figure S10**. Leave-One-Out Analysis for Return of Spontaneous Circulation. **Figure S11**. Influential Diagnostic Plot for Survival to Hospital Admission. **Figure S12**. Baujat Plot for Survival to Hospital Admission. **Figure S13**. Leave-One-Out Analysis for Survival to Hospital Admission. **Figure S14**. Influential Diagnostic Plot for Survival to Hospital Discharge. **Figure S15**. Baujat Plot for Survival to Hospital Discharge. **Figure S16**. Leave-One-Out Analysis for Survival to Hospital Discharge. **Figure S17**. Influential Diagnostic Plot for Shockable Rhythm. **Figure S18**. Baujat Plot for Shockable Rhythm. **Figure S19**. Leave-One-Out Analysis for Shockable Rhythm. **Figure S20**. Influential Diagnostic Plot for Medical Etiology of Out-of-Hospital Cardiac Arrest. **Figure S21**. Baujat Plot for Medical Etiology of Out-of-Hospital Cardiac Arrest. **Figure S22**. Leave-One-Out Analysis for Medical Etiology of Out-of-Hospital Cardiac Arrest. **Figure S23**. Influential Diagnostic Plot for Traumatic Etiology of Out-of-Hospital Cardiac Arrest. **Figure S24**. Baujat Plot for Traumatic Etiology of Out-of-Hospital Cardiac Arrest. **Figure S25**. Leave-One-Out Analysis for Traumatic Etiology of Out-of-Hospital Cardiac Arrest. **Data S1**. PRISMA-P 2020 Checklist.

## Data Availability

The datasets used and/or analyzed during the current study are available from the corresponding author on reasonable request.

## Abbreviations

COVID-19: Coronavirus disease 2019
SARS-CoV-2: Severe acute respiratory syndrome coronavirus 2
OHCA: Out-of-hospital cardiac arrest
CFR: Case fatality rate
TOR: Termination of resuscitation
ROSC: Return of spontaneous circulation
PRISMA: Preferred Reporting Items for Systematic Reviews and Meta-Analyses
PROSPERO: Prospective Register of Systematic Reviews
OR: Odds ratio
CI: Confidence interval
PPE: Personal protective equipment
EMS: Emergency medical services
